# Hepatitis C virus DNA vaccines: a systematic review

**DOI:** 10.1186/s12985-021-01716-8

**Published:** 2021-12-13

**Authors:** Ali Shayeghpour, Roya Kianfar, Parastoo Hosseini, Mehdi Ajorloo, Sepehr Aghajanian, Mojtaba Hedayat Yaghoobi, Tayebeh Hashempour, Sayed-Hamidreza Mozhgani

**Affiliations:** 1 Student Research Committee, School of Medicine, Alborz University of Medical Sciences, Karaj, Iran; 2 Department of Medical Virology, Tarbiat Modares University, Tehran, Iran; 3 Department of Virology, School of Public Health, Tehran University of Medical Sciences, Tehran, Iran; 4 Research Center for Clinical Virology, Tehran University of Medical Sciences, Tehran, Iran; 5 Hepatitis Research Center, Lorestan University of Medical Sciences, Khorramabad, Iran; 6 Department of Clinical Laboratory Sciences, School of Allied Medicine, Lorestan University of Medical Sciences, Khorramabad, Iran; 7 Department of Infectious Disease, School of Medicine, Alborz University of Medical Sciences, Karaj, Iran; 8 Shiraz HIV/AIDS Research Center, Institute of Health, Shiraz University of Medical Sciences, Shiraz, Iran; 9 Non-Communicable Diseases Research Center, Alborz University of Medical Sciences, Karaj, Iran; 10 Department of Microbiology, School of Medicine, Alborz University of Medical Sciences, Karaj, Iran

**Keywords:** Hepatitis C Virus, Immunogenicity, Systematic review, DNA vaccines, RNA vaccines, Immune response

## Abstract

**Background:**

Vaccination against HCV is an effective measure in reduction of virus-related public health burden and mortality. However, no prophylactic vaccine is available as of yet. DNA-based immunization is a promising modality to generate cellular and humoral immune responses. The objective of this study is to provide a systematic review of HCV DNA vaccines and investigate and discuss the strategies employed to optimize their efficacies.

**Methods:**

MEDLINE (PubMed), Web of Science, Scopus, ScienceDirect, and databases in persian language including the Regional Information Centre for Science & Technology (RICeST), the Scientific Information Database and the Iranian Research Institute for Information Science and Technology (IranDoc) were examined to identify studies pertaining to HCV nucleic acid vaccine development from 2000 to 2020.

**Results:**

Twenty-seven articles were included. Studies related to HCV RNA vaccines were yet to be published. A variety of strategies were identified with the potential to optimize HCV DNA vaccines such as incorporating multiple viral proteins and molecular tags such as HBsAg and Immunoglobulin Fc, multi-epitope expression, co-expression plasmid utilization, recombinant subunit immunogens, heterologous prime-boosting, incorporating NS3 mutants in DNA vaccines, utilization of adjuvants, employment of less explored methods such as Gene Electro Transfer, construction of multi- CTL epitopes, utilizing co/post translational modifications and polycistronic genes, among others. The effectiveness of the aforementioned strategies in boosting immune response and improving vaccine potency was assessed.

**Conclusions:**

The recent progress on HCV vaccine development was examined in this systematic review to identify candidates with most promising prophylactic and therapeutic potential.

## Background

Hepatitis C virus (HCV) particle contains a single‑stranded positive‑sense RNA genome that encodes a single polyprotein which is further processed to generate at least 11 polypeptides/proteins, including three structural proteins (core, and envelope proteins E1 and E2), a small polypeptide named p7, the novel F protein, and six nonstructural (NS) proteins (NS2, NS3, NS4A, NS4B, NS5A, and NS5B) [1, 2]. At present, infection with HCV poses a significant threat to global health and is associated with significant mortality and morbidity worldwide. Approximately, 184 million individuals were infected by the virus by 2005, many of which may progress to cirrhosis, liver fibrosis, and hepatocellular carcinoma if left untreated [3]. It was previously estimated that three to four million people are infected every year with about 90% being unaware of their chronic infection. This had led to projected estimations of up to 30 million carriers in china by 2050 if large-scale screening programs were not to be implemented [4, 5]. In line with this, almost all of 10 million chronic HCV carriers in Pakistan are unidentified, despite projection of a decreasing trend for the incidence of HCV infection [4, 6]. Therefore, considering the global burden of the infection, financial burden of direct-acting antiviral agents, risk of reinfection, and higher risk of hepatocellular carcinoma in previously infected individuals even after sustained virologic response [4, 7, 8], there is a strong incentive to develop prophylactic vaccines even with partial protection against HCV.

A novel vaccine for HCV would be able to significantly reduce the incidence of HCV infection and has the potential to achieve global control and possibly lead to the eradication of the virus. However, several barriers exists to development of such preventive measures including limited host tropism and full-length genome HCV culture in most cell lines, virus diversity, difficult identification of at-risk populations for testing vaccines, and the incomprehensive understanding of immune system and its protective response against HCV [9, 10]. Among the established vaccine types, a number of experimental nucleic acid-based vaccines are being developed which are mostly directed at inducing antibodies and cytotoxic T lymphocyte (CTL) responses against the non-structural proteins and envelope proteins of the virus.

A variety of elements have been implicated in adjusting the effectiveness of DNA vaccines such as host, target antigenic region, prime-boost approaches, presence or absence of adjuvant, dosage and immunization schedule which can be utilized to boost immunization outcomes [11]. To improve the effectiveness of the vaccines, several studies have been carried out to assess the efficacy of a variety of modifications in improving vaccine potency. In this regard, the use of truncated form of the highly immunogenic NS3 protein [12], taking advantage of vector-based and plasmids vaccines [13, 14], exploring prime-boost regimens with DNA and recombinant virus vaccines [15], utilizing multi-epitope DNA and peptide vaccines and novel techniques such as Gene Electrotransfer [16, 17], development of multigenotype vaccines [18], and inclusion of genetic adjuvants such as Human and avian hepatitis B virus (HBV) core antigen (HBcAg) [19], perforin (PRF) [20], heat shock protein gp96 [21], CC-chemokine ligand 20 (CCL20) gene [22], Interleukin-12 (IL-12) [23], IL-23, granulocyte-monocyte colony stimulating factor (GM-CSF) [24], etc. have been explored in previous studies.

In this study, a systematic review of the existing literature over the last 20 years was conducted to assess the efficacy, effectiveness, immunogenicity, and safety data regarding HCV nucleic acid vaccines on laboratory animals. We have also outlined a number of approaches to improve vaccine efficacy, in hopes that this work will aid future studies in development of prophylactic and therapeutic vaccines against HCV.

## Methods

### Search strategy

This study reviewed published articles in English and Persian language between 2000 and 2020. International databases including MEDLINE (PubMed), Web of Science, Scopus and ScienceDirect as well persian repositories including the Regional Information Centre for Science & Technology (RICeST), the Scientific Information Database and the Iranian Research Institute for Information Science and Technology (IranDoc) were explored to find relevant reports on HCV nucleic acid vaccines from 2010 to 2020. The keywords and terms used to find articles relevant to DNA immunization included ‘Hepatitis C Virus and DNA vaccine’, ‘HCV DNA vaccine and development’, ‘HCV DNA vaccine and Adjuvant’, ‘HCV DNA vaccine and Antigenic target’, ‘HCV DNA vaccine and Immunogenicity’, ‘HCV DNA vaccine and efficiency’. For RNA-based immunization the following keywords were used: ‘Hepatitis C Virus and RNA vaccine’, ‘Hepatitis C Virus and RNA and vaccine’, ‘HCV RNA vaccine and development’, ‘HCV RNA vaccine and Adjuvant’, ‘HCV RNA vaccine and Antigenic target’, ‘HCV RNA vaccine and Immunogenicity’, and ‘HCV RNA vaccine and efficiency’.

In addition, relevant cited references in the original articles were examined to include articles which were not indexed by the aforementioned databases. References and their abstracts were saved and reviewed using EndNote X9.1 and EndNote 20 reference manager software (Clarivate Analytics, USA).

### Selection criteria and data extraction

Following systematic search, studies were screened to select original research articles focusing on HCV infection, HCV DNA vaccines and their immunogenicity and efficacy. Reports were removed if the individuals in the studies were infected with other genera of hepaciviruses or if the study was based on non-DNA vaccines. Studies without full texts or in languages other than persian or english were also excluded.

Two independent reviewers screened and evaluated the included studies to extract the following data: title, abstract, main text, authors, country of origin, the host, target antigenic region, number of boosters, presence or absence of adjuvant, dosage, and immunization.

## Results

**Fig. 1:**
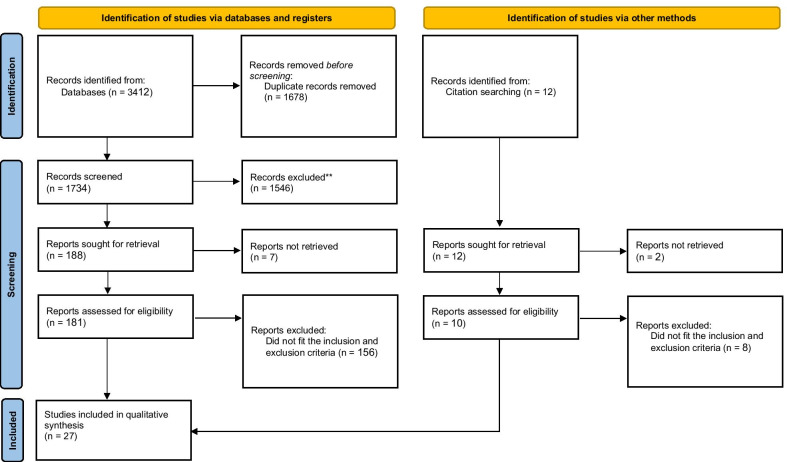
Preferred Reporting Items for Systematic Reviews and Meta-Analyses (PRISMA) flow diagram for search and screening strategy

The preliminary search for DNA-based vaccine development identified 853 papers in Web of Science, 474 papers in MEDLINE (PubMed), 1381 papers in Scopus, 678 papers in ScienceDirect, 38 papers in Iranian databases, and 12 articles from manual search. After omission of 1678 duplicate papers, 1758 articles were surveyed for eligibility, out of which 27 articles were included. The search for RNA vaccines yielded no relevant studies, alluding to the lack of research on RNA vaccines for HCV. Therefore, a total of 27 records were found eligible for this systematic review (Fig. 1). A summary of the prominent included studies is available in Table 1.

**Table 1:** A summary of studies on DNA-based vaccines for HCV and their efficiency in animal models

Row	Author(s)	Year	Host	Genomic region for design	Route	Prescribed number	Adjuvant	Laboratory method	Efficiency	References
1	Pouriayevali, M. H., et al	2019	BALB/c mice	NS3 gene sequence (1095-1380aa)	ID	three doses in two-week intervals	Listeriolysin O (LLO)	PCR, western blot, lymphocyte proliferation, cytotoxicity, and cytokine levels assays	Enhancement of total IgG in mixed responses with Th1 dominancy	[56]
**2**	Lee, H et al	2017	BALB/c mice	NS3 to NS5A	IM	three doses in two-week intervals	murine IL-28B	Body weight change, necropsy, hematological and serum biochemical evaluation, LD50 determination, IFN-γ ELISpot assay	Increased NS3,4, and 5 IFN-γ spots in ELIspot assay Murine IL-28B resulted in more than twice NS3/4A specific IFN-γ spots per million splenocytes	[75]
3	Pouriayevali, M. H., et al	2016	BALB/c mice	NS3 gene (1095—1379 aa)	ID	four doses in two-weeks intervals	N/A	RNA Extraction and cDNA Synthesis, total and subtypes of IgG antibody assay, cell proliferation assay, western blot and ELISPOT	induced significant levels of total antibody, IgG2a, IFN-γ and IL-4	[12]
4	Levander, S., et al	2016	C57BL/6 J mice	NS3/4A-stork-HBcAg	IM	one to five times with doses of 50, 5, 0.5, 0.05, and 0.005 µg	HBcAg	In vitro transcription and translation assay, Transient transfection, western blot and ELISpot assay	Increased production of IFN-γ, IL-2 and	[19]
5	Behzadi, M. A., et al	2016	C57BL/6 mice	NS3/NS4A	IM	Three injection, 2 weeks apart	Freund’s adjuvant, MPL	flow cytometry, T helper frequency using cell staining	Increased production of Th1/ Th2 and T-CD8 + cells	[26]
6	Yazdanian, M., et al	2015	BALB/C mice	HBsAg and core (amino acids 2–120)	IV	Three doses	pluronic acid (F127)	CTL assay, SDS–PAGE and western blotting for plasmid expression, Antigen specific proliferation assay, and ELISpot	shifting the immune responses pathway to Th1 by enhancing the IFN‑γ cytokine level	[1]
7	sun, w.,et al	2015	BALB/C mice	HCV E2 with an immunoglobulin Fc fusion tag	SC	Approximately Four doses in two-weeks intervals	CpG ODN/Quil A	ELISA assay, Lymphocyte Proliferation Assay, Immunofluorescence Assay and HCVpp Neutralization Assay	Enhancement of E2-specific humoral and cellular immune response	[46]
8	Pishraft Sabet, L., et al	2015	CB6F1 mice	Core(132–142),, E2(412–426), NS3(1073–1081)(1248–1262) and NS5B(2727–2735)	IM	three times at 2-week intervals,	heat shock protein gp96 (NT(gp96))	flow cytometry and ELISA	Induction of T-cell and antibody responses	[21]
9	Masalova, O. V., et al	2015	DBA/2 J mice	NS3–NS5B	IM	three times with an interval of 2 weeks	pcGM–CSF	ELISA, ELISpot	Enhancement of humoral and cellular immune response	[32]
10	Gummow, J., et al	2015	C57BL/6 mice	nonstructural (NS) proteins (3, 4A, 4B, and 5B)	ID	either two or three doses at 2-week intervals	perforin	Cell culture., ELISpot, T-cell proliferation assay, Flow cytometry and Western blot	Enhancement of TNF-a-producing CD4? and CD8? T-cells	[50]
11	Hartoonian, C. et al	2014	BALB/C mice	Core protein	SC	three times in 2 weeks intervals	Macrophage Inflammatory Protein 3-beta (MIP-3beta)	ELISpot and cytotoxic Granzyme B release assays), ELISA	Enhancement of IFN-γ/ IL-4 and anti-core IgG2a/IgG1 ratio, lymphoproliferation, strong cytolytic GrzB release	[55]
12	Hartoonian, C. et al	2014	BALB/c mice	Core protein	SC	three times in 2 weeks intervals	CC-chemokine ligand 20 (CCL20)	ELISA/ELISpot and cytotoxic Grenzyme B (GrzB) release assays	Enhancement of core specific IFN-c/IL-4 ratio, IL-2 release, IFN-c and the core-specific serum total IgG and IgG2a/IgG1 ratio were significantly higher when the pCCL20 was co-inoculated	[22]
13	Ahlen, G. et al	2014	BALB/c (H-2d) and/or C57BL/6 J (H-2b) mice	NS3/4A	gene gun delivery to the skin or by IM	one, two, three or four times at monthly interval	N/A	ELISpot, RMA-S stabilization assay and cytotoxicity assay	Production of NS3/4A-specific T cells in vitro	[76]
14	Gorzin, Z. et al	2013	C57BL/6 mice	non-Structural Protein 2 (NS2)	IM	three times with an interval of 2 weeks	IL-12	ELISA, Cytokine secretion assay, MTT	Enhancement of CTL response, interferon-γ production, and lymphocyte proliferation compared to negative control	[77]
15	Wada, T. et al	2013	C57BL/6 mice	structural protein (CN2), non-structural protein (N25)	IP	three times at 48-h intervals	N/A	cytotoxicity assay, ELISPOT assay, Generation of CTL effector cells and cytotoxicity assay, Histopathological examination and ELISA	significant decrease in the expression of HCV protein in mice administered the N25 DNA vaccine and improvement of pathological changes in the liver by N25 DNA vaccine	[78]
16	Naderi, M. et al	2013	C57BL/6 mice	NS3	IM	Three times	IL-12	RT-PCR, western blot, ELISA, Lymphocyte proliferation assay	production of significant levels of both IL-4 and interferon (IFN)-γ and enhancement of cytotoxicity and lymphocyte proliferation responses of vaccinated mice	[23]
17	Holmstorm, F. et al	2013	C57BL/6 J mice	Optimized Synthetic Codon- of NS5A	IM	One to three times at monthly intervals	N/A	ELISA, western blot, ELISPOT assay, cytotoxicity assay and RMA-S stabilization assay	Production of high Ab levels, IFN-γ and lytic cytotoxic T cells	[79]
18	Park, S. et al	2010	monkeys as the naive group	E2	IM	6 times at the indicated months	IL-7	ELISA, ELISPOT assay	Enhancement of antibody responses specific for HCV E2 protein and specific T cell responses by codelivery of hIL-7 DNA	[80]
19	Masalova, O. V. et al	2010	Female DBA/2 J mice	Full-size NS5A protein	IM	thrice with a month’s interval	Immunomax	Eukaryotic Cell Transfection, ELISA	Enhancement of cellular immune response, secretion of antiviral cytokines IFN-γ and IL-2	[81]
20	Martin, P. et al	2008	HLA-A2 transgenic mic	NS3, NS4 and NS5B proteins	IV	twice, 2 weeks apart	N/A	ELISpot and CTL assays	Induction of strong and broader IFN-γ ELISpot and CTL responses	[13]
21	Long, K. L. et al	2008	C57BL/6 mice and monkey	NS3/NS4A	IM	Three times, 2 weeks apart in mice and two times, 4 weeks apart in the monkeys	N/A	Immunofluorescenc, ELISpot,	Induction of strong anti-NS3/NS4A T cell responses	[82]
22	Folgori, A. et al	2006	Chimpanzees (Pan troglodytes)	NS3- NS5B region	IM	Adenovirus injection: Two times, 4 weeks apart administered bilateraly, booster injected at week 25. plasmid DNA injection at 35, 37,39 weeks	N/A	IFN-γ intracellular staining, cytotoxicity assay	High CMI in 4 out of 5 subjects, no increase of hepatic enzymes in vaccinated chimpanzees. HCV specific intrahepatic CD8 + T-cell response	[83]
23	LI, Y. P. et al	2006	B6C3F1 mice and piglets	E2	ID and SC	Three times, 3 weeks apart	N/A	CTL assay and ELISA	Strong stimulation of strong Th1-like immune responses in mice, more balanced immune responses in piglets, production of higher E2-specific antibody levels and shifting the immune response towards Th2-like ones in piglets	[84]
24	Ahlen, G. et al	2005	wild- type or CD8-/- C57BL/6 mice	NS3/4A	IV	Two times, 2 weeks apart	N/A	SDS PAGE and Western blot	Enhancement of T specific cells by transdermal DNA-based vaccination entered the liver and Clearance of NS3/4A-expressing hepatocytes in transiently trans- genic CD8 + / + mice but not in CD8^−^/^−^ mice	[85]
25	Zhu, M. et al	2004	BALB/c mice	E1/E2	quadriceps muscles	Three times, two weeks apart	CpG	HCV antigen specific proliferation assays and cytokine secretion assays	Enhancement of humoral and cellular immune responses. CpG adjuvant significantly enhances the cellular immune response	[86]
26	Ou-Yang, P. et al	2002	BALB/c mice	Core protein	IM	Two times, seven days apart	GM-CSF	ELISA, Immunofluorescence, proliferative assay	Higher Antibody titer and cytotoxic T cell and protein expressing CD11c^+^ Dendritic cells	[87]
27	Ma, X. et al	2002	BALB/c (H-2d) mice	E2	IM	Three times with a month’s interval	CpG	ELISA and CTL assay	superior antibody and CTL responses	[88]

ID; intradermally, IM; intramuscularly, IV; intravenously, MPL; monophosphoryl lipid A, SC; sub-cutaneously, IP; intraperitoneally

### HCV vaccine target sequence

#### Protein selection

Previous studies have focused on several viral proteins and their combinations in generating adequate immune response for HCV vaccine development. Among the proteins assessed for vaccine utilization, plasmids encoding HCV NS3, NS4, NS5, core, and the envelope proteins E1/E2 as their full or truncated isoforms, or fractured peptides consisted the majority of the previous works. A summary of these studies is provided below.

The expression of partial length of NS3 gene, which encodes immunogenic epitopes (1095–1379 aa) was demonstrated to be capable of induction of significant levels of total antibody, IgG2a subclass antibody, Interferon (IFN)-γ, Interleukin (IL)-4 and proliferation assay [12]. Furthermore, incorporation of NS4A in NS3-based genetic immunization using a cloned full-length genotype NS3/4A gene resulted in increased expression of NS3 and higher levels of NS3-specific antibodies (10- to 100-fold) and IgG2a/IgG1 ratio (420 vs. 3) in humoral responses compared to NS3 gene, demonstrating a favorable Thelper 1-skewed response. In line with this, low dose i.m. (10 mg) immunization with the NS3/4A gene inhibited the growth of an NS3/4A-expressing tumor cells in vivo, whereas immunization using the NS3 gene alone or NS3 protein did not. Furthermore, a three to four 4 mg dose of a NS3/4A plasmid administed using a gene gun primed CTL at a precursor frequency of 2–4%, which inhibited the growth of NS3/4A- expressing tumor cells in vivo. further demonstrating the effectiveness of multiprotein DNA vaccines [25]. The direct immunogenicity of NS3/4A plasmids were also examined by Ahlen et al. and Behzadi et al. whom reported removal of transiently transgenic hepatocytes by T cells in liver and significant cell-mediated immune responses with a DNA vaccine encoding HCV-3a NS3/NS4A in C57BL/6 mice, respectively [26, 27].

The safety concerns regarding RNA helicases and their disruption of cellular functions and their possible role in development of cancer had let Ratnoglik et al. construct plasmids encoding mutated NS3 protein without serine protease and helicase catalytic activity to replace their bioactive counterpart in vaccine development. They subsequently demonstrated comparable immune response following immunization using non-catalytic NS3 mutants compared to wild type NS3. Their results indicated that the comparable effectiveness and reduced bioactivity of these immunogens, would prove to be efficient alternatives to wild type HCV proteins in DNA vaccines against HCV [28]. Gene electrotransfer of a DNA vaccine encoding an optimized version of the nonstructural region of HCV (from NS3 to NS5B) also induced substantially more potent, broad, and long-lasting CD4+ and CD8+ cellular immunity than naked DNA injection in mice and in rhesus macaques, as measured by a combination of assays, including IFN-γ ELISPOT, intracellular cytokine staining, and CTL assays [17]. Furthermore, the work of Martin, P et al. revealed three epitopes mapping within the NS3 protease (GLL: aa 1038–1047) or helicase (ATL: aa 1260–1268 and TLH: aa 1617–1625) which display similar and high in vitro binding capacities to soluble HLA-A2 molecules, are capable of inducing either CTLs and/or IFN-γ-producing T cells. This peptide could recall in vitro HCV-specific IFN-γ and IL-10-producing T cells from peripheral blood mononuclear cells of chronically infected patients. These data increase the pool of NS3-specific CD8 + T-cell epitopes available to analyze HCV associated immunity and could contribute to the design and evaluation of candidate vaccines [29].

It is of note to say to that in vaccinations of chimpanzees with recombinant DNA and adenovirus expressing HCV core, E1E2, and NS3-5 genes based on HCV1b genotype, viral loads were 100 times lower than naïve infected controls and the antibody levels against E2 glycoproteins were inversely correlated with peak viral loads after intravenous challenge. Interestingly, one vaccine that had sterilizing immunity against slightly heterologous virus, generated highest level of E2-specific total and neutralizing antibody responses as well as NS3/NS5-specific T-cell proliferative responses. The other four vaccinees with low levels of E2-specific antibody had about 44-fold reduced peak viral loads but eventually developed persistent infections [30].

Other studies have focused on using DNA-based vaccines utilizing multiple expression cassettes. Expression of HCV core protein regulated by an inducible in vivo-activated Salmonella promoter and HCV E2 protein by the cytomegalovirus enhancer/promoter resulted in efficient induction of HCV core and E2-specific cellular immune responses and antibodies in oral immunization of BALB/c mice with the attenuated Salmonella strain SL7207 carrying this plasmid. IgG purified from immunized mice could neutralize the infectivity of HCV pseudoparticles of both the autologous Con 1 isolate and the heterologous H77 isolate, and cell culture-produced HCV of Con1-JFH1 chimera [31]. While using multiple expression cassettes stands out as a promising modification to plasmid sequence for boosting immunity, further studies are needed to confirm their efficacy and safety.

#### Multi-epitope expression

Previous studies have shown that immunization using serial administration of multi-epitope plasmid DNAs and peptides harboring immunodominant CD8 + T cell epitopes (HLA-A2 and H2-Dd) from Core (132–142), NS3 (1073–1081) and NS5B (2727–2735), a Th CD4 + epitope from NS3 (1248–1262), and a B-cell epitope from E2 (412–426)) formulated with Montanide ISA 720 adjuvant resulted in less robust immune response and HCV-specific level of total IgG, IgG1 and IgG2a in those receiving heterologous DNA/peptide and DNA/DNA compared to those receiving peptide/peptide regimen. Interestingly, IFN-γ levels in those receiving three doses of the peptide vaccines were significantly higher than those receiving two doses of the plasmid vaccine and a single dose of the peptides with Montanide ISA 720 adjuvant. Notably, the triple-dose peptide vaccine had a higher IFN-γ/IL-4 ratio compared to the other group.weeks [16]. In contrast, Masalova et al. have demonstrated that combined use of a fragmented NS3-NS5B plasmid and recombinant core, NS3, NS4, NS5A, and NS5B proteins and peptides induced statistically greater cytokine expression compared to recombinant proteins and plasmid-based immunogens alone. Furthermore, their findings demonstrated that the pcNS3–NS5B plasmid induced a more T-cell-oriented response, whereas the recombinant NS3 and NS5B proteins stimulated a potent humoral immune response [32].

Moreover, mice receiving polycistronic construct capsid/E1/E2/NS2/NS3 (pRC/C-NS3) encoding 5 structural and nonstructural proteins in a canarypox virus had enhanced antibody and cellular responses to HCV proteins. Immunodominant CD8 + T cell responses to several HCV structural and nonstructural proteins, characterized by cytotoxicity and interferon-γ production or IFN-γ production without significant cytotoxicity, were observed in both strains of mice. The combination of naked DNA with a non-replicating canarypox booster encoding HCV polycistronic pRC/C-NS3 genes appears to diversify and enhance T-cell responses to HCV proteins [33].

A multiple antigenic peptide immunization using six peptides derived from conserved epitopes in E1, E2 (n = 2), NS4B, NS5A and NS5B administered to BALB/c mice as HCVp6-multiple antigenic peptide at doses ranging from 800 ng to 16 μg resulted in humoral responses to structural epitopes, induction of IFN-γ producing CD4+/CD8+ T- lymphocytes at extended durations i.e., > 20 weeks, and viral neutralization of genotypes 2a and a chimeric 2a/4a virus in HCV culture using mice sera at > 1600 ng/animal doses for at least 20 weeks. They have shown that HCVp6-multiple antigenic peptide confers potent viral neutralization and specific cellular responses [34].

Different combinations of a candidate HCV vaccine consisting of 100 µg recombinant HCV core/E1/E2 DNA plasmid and/or 25 µg recombinant HCV polyprotein and 50µL Montanide ISA- 51 were also constructed in a previous study. IgG titers for specific HCV antibodies (total IgG, IgG1, and IgG2a), cell proliferation, and intracellular IFN-γ 4 weeks after the final injection was only assessed in mice immunized with recombinant HCV DNA plasmid, recombinant HCV polyprotein, and montanide and mice immunized with recombinant HCV polyprotein and montanide. IgG1 was the predominant antibody detected in the group receiving recombinant HCV DNA and HCV core/E1/E2 polyprotein, and montanide as well as those receiving recombinant polyprotein and montanide. However, no IgG2a response was detected in any of the groups. Proliferation assays demonstrated that splenocytes from recombinant HCV DNA primed/recombinant HCV polyprotein boost group had developed significant anti-HCV proliferative responses. The combination of a recombinant HCV DNA plasmid, recombinant HCV polyprotein, and montanide induced a high antibody titer with a predominance of IgG1 antibodies while recognizing the major neutralization epitopes in hypervariable region 1 of E2 glycoprotein [35].

BALB/c mice (H-2d restricted) vaccinated intramuscularly with a multi-CTL epitope gene consisting of two epitopes of HCV (H-2d-restricted HCV core 133–142 and E1315–322) cloned into the eukaryotic expression vector pcDNA3.1 inducted CTLs against target cells (P815, H-2d restricted) pulsed with various CTL epitope peptides. Therefore, the multi-CTL epitope-based DNA vaccine directed against two HCV CTL epitopes could induce specific CTL responses to each of the two CTL epitopes independently and long-term CD8+ T-cell memory responses. The epitope-specific CTLs produced helper T-cell type 1 cytokines. [36].

Furthermore, chimpanzees immunized transdermally twice with recombinant replication competent vaccinia viruses expressing HCV genes resulted in resolution of HCV infection with the rate of chronicity between the immunized and the control animals being close to statistical significance (*P* = 0.067). Immunized animals developed vigorous IFN-γ enzyme-linked immunospot responses and moderate proliferative responses [37]. To investigate cross-genotype protection, the immunized, recovered chimpanzees in the described study were challenged with a pool of six major HCV genotypes. During the acute phase after the multigenotype challenge, all animals had high-titer viremia in which genotype 4 dominated (87%), followed by genotype 5 (13%). After fluctuating low-level viremia, the viremia eventually turned negative or persisted at very low levels [37].

#### Optimization of prime-boost regimen

Vaccines designed as prime-boost regimens confer further protection by repeated and/or serialized exposure of viral epitopes during immunization period. Generally, the most successful protocol for DNA immunization to induce CTL responses is priming plasmid DNA to induce low-level, persistent immunity with strong but short-lived immunity of the recombinant virus boosters [38, 39]. However, the use of heterologous DNA prime–recombinant adenovirus boost regimen also had promising results.

Mice primed with either conventional pVRC-based or suicidal pSC-based DNA vaccines carrying DEC-205-targeted NS3 antigen and boosted with type 5 adenoviral vectors encoding the partial NS3 and core antigens C44P, induced a marked increase in antigen-specific humoral and T-cell responses in comparison with either recombinant Adenovirus-based vaccines or DEC-205-targeted DNA immunization in isolation. The protective effect against heterogeneous challenge was correlated with high levels of antiNS3 IgG and T-cell-mediated immunity against NS3 peptides. Moreover, prime-boosting with a suicidal DNA vaccine (pSC-DEC-NS3), which elicited increased TNF-a-producing CD4+ and CD8+ T-cells against NS3-2 peptides (aa 1245–1461), demonstrated increased heterogeneous protection compared with priming with a conventional DNA vaccine (pVRC-DEC-NS3) [40].

Another study reporting the development of recombinant Lambda bacteriophage nanoparticles encoding HCV core antigen investigated the antigen-specific immune responses triggered in mice by different prime–boost combinations. The homologous prime/boost with recombinant Lambda nanoparticles induced higher levels of cellular and humoral immune response than the DNA vaccines. However, the protective effects of the vaccine (lymphocyte proliferation, CD8+ cytotoxic activity) was still lower than a heterologous prime immunization with HCV Core protein using DNA vaccine followed by Lambda boost, which resulted in shifting the immune response toward a Th1 pattern with a greater overall immunity [41]. A DNA-based vaccine expressing HCV genotype 1a NS3/4A proteins and a boost regimen with a modified vaccinia virus expressing genotype 1b NS3/4/5B proteins (MVATG16643) also induced significantly higher levels of IFN-γ or IL-2 ELISpot responses compared with each vaccine alone, independent of the time of analysis and the time interval between vaccinations. Both CD8+ and CD4+ T-cell responses as well as the spectrum of epitopes recognized were improved. A significant increase in polyfunctional IFN-γ/tumor necrosis factor α (TNF-α)/CD107a^+^ CD8+ T cells were detected following vaccination (from 3 to 25%), and prime/boost was the only regimen that activated quadrifunctional T cells (IFN-γ/TNF-α/CD107a/IL-2). Incorporation of interleukin-12 (IL-12) expression in DNA plasmid also led to a highly efficient CTL induction and clearance of HCV-core expressing vaccinia virus in a triple prime-boost regimen using HCV core antigen [38]. In vivo functional protective capacity of the DNA prime/MVA boost was also demonstrated in a Listeria-NS3-1a challenge model [42].

Noticeably, recombinant adenovirus vaccines elicited greater levels of IFN-γ secreting T-cell response and CTL response than the DNA vaccines in a study by Park, S. H et al.. However, a heterologous regimen priming DNA vaccine with a recombinant resulted in higher level of Th1 responses compared to the other regimens including double immunuzation with the recombinant adenovirus. Furthermore, three E2-specific CTL epitopes were mapped using a peptide pool spanning the E2 protein sequence (a.a. 384–713) in BALB/c mice, and one of these (E2 405–414: SGPSQKIQLV) was shown to be immunodominant. It is of note to say that no significant differences were found in the repertoire of E2-specific T-cell responses or in the immunodominance hierarchy of the three epitopes with different regimens, demonstrating the effectiveness of the heterologous DNA prime-recombinant adenovirus boost in confering T-cell based immune response[43]. Triple prime-boost immunization combination with HCV core expression using plasmid (pCEP4-core) and replication- defective adenovirus (Adex1SR3ST) in another study induced CTL response in mice in all but one combination in which all three immunizations were done using the plasmid vaccine [38].

### Complementary strategies

While a considerable portion of the reviewed literature on HCV vaccine development focused on optimal protein and epitope selection and prime-boost combinations, a group of studies have also focused on strategies to refine adjuvants, target sequences, and vaccine delivery which could be used in tandem with the aforementioned strategies in order to boost or prolong vaccine immunogenicity.

### Adjuvant selection

Previous reports have studied a variety of potential adjuvants for HCV vaccines. For instance, Levander et al. research showed that HBV core antigen (HBcAg) can act as an adjuvant in hepatitis C virus (HCV) based DNA vaccines. Addition of full-length and fragmented stork HBcAg gene sequences added to an HCV non-structural 3/4A protein gene (NS3/4A-stork-HBcAg) resulted in an enhanced priming of HCV-specific IFN-γ and IL-2 responses in both wild-type and NS3/4A transgenic mice, the latter with dysfunctional NS3/4A-specific T-cells [19]. On the other hand, Behzadi et al. have shown in their study that the use of complete Freund’s adjuvant, monophosphoryl lipid A can induce the production of Th1/Th2 cells and CD8+ T-cells in all the immunized groups [26]. Interestingly, an eccentric study on vaccine development have shown that polysaccharides derived from Artemisia annua could be utilized as an adjuvant and modulate immune response induced by a NS3 plasmid DNA vaccine by increasing IFN-γ but not antibody or IL-4, suggesting the mechanism to be the result of modulation of Th1 response [44]. Synthesized Poly(D,L-lactic acid)-co-poly(ethylene glycol)-co-poly(D,L-lactic acid) (PLA–PEG–PLA) and poly(d,l-lactic-co-glycolic acid;PLGA)–PEG–PLGA used as micelles with encapsulated plasmid pcDNA3.1 also had appealing results as adjuvants with no considerable side effects in single oral immunization with DNA/polymers with noticable immune responses in in vivo tests [45].

### Heat shock protein gp96

Utilizing N-terminal domain of heat shock protein gp96 (NT(gp96)) has also been shown to be a potent adjuvant for enhancing immunity. A PT DNA vaccine studied in a previous study which encoded four HCV immunodominant CTL epitopes (two HLA-A2- and two H2-Dd-specific motifs) from the Core, E2, NS3 and NS5B antigens in addition to a T-helper CD4 + epitope from NS3 and a B-cell epitope from E2 with the NT(gp96) was fused to the C- or N-terminal end of the PT DNA (PT-NT(gp96) or NT(gp96)-PT) demonstrated that immunization of mice with PT DNA vaccine fused to NT(gp96) induced significantly stronger T-cell and antibody responses than PT DNA alone. Additionally, the adjuvant activity of NT(gp96) was more efficient in the induction of immune responses when fused to the C-terminal end of the HCV DNA polytope [21].

### HBsAg and immunoglobulin Fc

Immunization with a vector harboring coding sequence of HBsAg and Hepatitis C Virus core protein in tandem within the pCDNA3.1 backbone shifted the immune responses pathway to T-helper (Th1) by enhancing the IFN‑γ cytokine level much higher than protein immunization while the proliferative and CTL responses were comparatively the same (or slightly in favor of DNA immunization) [1]. Moreover, a recombinant plasmid termed cDNA3.1-E2-Fc expressing HCV E2 with an immunoglobulin Fc fusion tag induced higher titers of E2-specific IgG in mice immunized with pcDNA3.1-E2-Fc compared to mice immunized with pcDNA3.1-E2 alone. Furthermore, pcDNA3.1-E2Fc immunization could boost E2-specific lymphocyte proliferation and enhance the secretion of IFN-γ by lymphocytes upon in vitro stimulation with soluble E2 compared to pcDNA3 [46].

Immunization of mice using several HCV epitopes (encoding; core132–142 [C], E2405–414 [E4], E2614–622 [E6] and NS31406–1415 [N] CD8+ CTL epitopes as CE4E6N polytope) and its HBsAg-fused counterpart elicited strong CTLs and IFN-γ-secreting cells that were further augmented towards a Th1 response and partial tumor protection by DNA-prime/peptide-boosting regimen compared to the adjuvant-formulated synthetic-peptide immunization. Priming with HBsAg alone could not explain the augmenting effect of the vaccine, indicating the importance of priming by polytope itself.

Several other studies have also investigated the modulation of immune response against HCV immunogens using HBV antigens. Co-Administration of DNA Vaccine Encoding HBV Surface Antigen and HCV Envelope Antigen in BALB/c mice resulted in antibody production against both HBV and HCV and increased expression of IL-2 and RANTES but not IL-4, and therefore, inducing a Th1 response [47]. Furthermore, immunization of C57BL/6 mice with plasmid DNA expressing five fragments of HCV E2 fused to HbsAg gene was accompanied by an IgG2a-dominant antibody production against both proteins in mice sera and high IFN-γ expression in cultured splenic cells [48]. Hence, fusion of immune carriers like HBsAg conjoined with DNA-prime/peptide-boost immunization regimen is a feasible strategy to enhance the epitope-specific immune responses towards poly-CTL-epitopic vaccines [49].

### Perforin

Coexpression of PRF, a pore forming protein released by CD8 T-cells and NK cells, and HCV antigens on a single plasmid elicits strong cell mediated immunity against the HCV NS proteins (3, 4A, 4B, and 5B) [50]. The results of the same study evaluating the vaccine demonstrated that encoding the NS4A protein in a vaccine which encoded only NS3 counterintuitively reduced the immunogenicity of NS3, while addition of PRF increased NS3 immunogenicity. On the other hand, the inclusion of NS4A in a PRF-encoding DNA vaccines increased the immunogenicity of the NS3, NS4B, and NS5B proteins [50]. Furthermore, a truncated mouse PRF with cytolytic activity lacking the final 12 amino acids of the C terminus was used instead of the full-length protein [51], which was also determined to be more immunogenic than the respective canonical DNA vaccine lacking PRF. DNA-based vaccines using pNS3-PRF, pNS4/5-PRF, pNS3/4/5B plasmids could elicit robust long-term cell mediated immunity evidenced by high reactogenicity with Interferon-γ enzyme-linked immunosorbent spot (ELISpot) assay against HCV peptides. The multi-antigenic vaccine primed with PRF promoted cytolytic vaccination without any adverse side-effects in mice [52]. The mechanisms for the higher immunogenicity generated by PRF could be explained by the study conducted by Grubor-Bauk et al. They examined the mechanism of cell death by the bicistronic DNA vaccine encoding the HCV NS3 and PRF under the control of CMV and SV40 promoters, respectively. The results of the study showed that the inclusion of perforin in the DNA vaccine altered the fate of NS3-positive cells from apoptosis to necrosis, and resulted in more robust immune responses in mice and pigs [20].

### GM-CSF

The conflicting results of the previous studies, precludes decisive judgement on inclusion of GM-CSF in HCV DNA vaccines. In contrast to some of the previous works such as the one from Masalova O. V., et al. who showed pcGM-CSF increase humoral and cellular immune response [32], Chen et al. demonstrated that co-inoculated GM-CSF causes significant suppression to the dengue virus type 1 and type 2 prM-E DNA vaccinations and influences protective efficiency against virus challenge. Counterintuitively, GM-CSF showed little or no effect on the immune response elicited by hepatitis C virus C or E1 DNA vaccine candidates. Notably, these effects of GM-CSF were long-lasting [53]. Moreover, co-vaccination of DNA encoding GM-CSF and Flt-3 ligand together with a plasmid encoding for the HCV NS5 protein induced increased antibody responses and CD4 + T cell proliferation to this protein. Vaccination with DNA encoding GM-CSF and Flt-3L promoted protection against tumor formation and/or reduction in mice co- immunized with cytokine-encoding DNA constructs [54].

### Macrophage inflammatory protein 3-beta

Macrophage inflammatory protein 3-beta gene added as an adjuvant in a triple-dose HCV core DNA vaccine elicited an enhanced Th1 biased systemic immune response as evidenced by higher IFN-γ/ IL-4 and anti-core IgG2a/IgG1 ratio, lymphoproliferation, strong cytolytic GrzB release and enhanced population of IFN-γ producing immunocytes. Likewise, the humoral immune response assumed as the total anti-core IgG level was augmented by Macrophage inflammatory protein 3-beta co-delivery [55]. Similarly, mice co-immunized with a CCL20-containing plasmid developed higher levels of core specific IFN-γ/IL-4 ratio and IL-2 release, IFN-γ producing cells, lymphocyte proliferation and cytotoxic Granzyme B release in both draining lymph nodes and spleen cells of immunized mice. The core-specific serum total IgG and IgG2a/IgG1 ratio were significantly higher when the pCCL20 was co-inoculated [22].

### Listeriolysin O

Pouriayevali et al. introduced Listeriolysin O of Listeria monocytogenes (toxin with an extremely immunogenic feature) as an attractive adjuvant. They have shown that introduction of NS3 and Listeriolysin O gene induced the highest titer of total IgG against NS3 antigen compared with other controls. Determination of IgG subclasses confirmed an effective increase in mixed responses with Th1 dominancy. In addition, significant levels of cytokines (*P* < 0.05) and lymphocyte proliferation responses (*P* < 0.05) indicated the superiority of this regimen. The findings may have important implication for Listeriolysin O gene application as genetic adjuvant in immune response against HCV [56].

### IFN-λ3

Addition of IFN-λ3 to a plasmid encoding NS3-NS5A also increased IFN-γ spot-forming cells and both CD4+ and CD8+ T cell subsets produced multiple cytokines. However, the frequency and phenotype of HCV-specific MHC-I dextramer + CD8+ T cells were not changed. Interestingly, the frequency of Treg cells, particularly activated Treg cells, was decreased which was in line with previous reports indicating that IFN-λ3 adjuvants decrease Treg cell frequency. Ex vivo IFN-λ3 treatment similarly reduced Treg frequency in pre-vaccination peripheral blood mononuclear cells. Finally, Treg cell frequency inversely correlated with HCV-specific, IFN-γ-producing T cell responses in the study participants [57].

### Modification of signal sequence

Injection of plasmids encoding full-length E2 and nonstructural protein 1 (p7) fused to either 13 or 38 C-terminal amino acids (aa) of HCV E1 or a complete E1 stop-transfer signal with duplicated second hydrophobic segment resulted in potent antibody production against E2/p and T-helper cell response targeted against hypervariable region 1, aa 472–586 of E2, and a novel epitope at aa 774–796 of p7. Profile of cytokines secreted by proliferating mouse splenocytes stimulated in vitro with E2- and p7-derived peptides, indicated mixed Th1/Th2 type of immune response. Thus, the full-length E2 and p7 genes supplied in one cassette modulated the immunogenic profile of E2. E2/p7 containing a complete E1 stop-transfer signal with prolonged membrane spanning domain was superior to the shorter E2/p7 version in terms of both antibody and cellular immunogenicity. Thus, optimal performance of HCV E2 could be achieved through modification of the E2 signal sequence, the release of E2 from the rough ER while retaining full-length E2 and p7 sequences, improving Th2 performance of HCV envelope genes as prototype vaccine [58].

### CpG adjuvants and plasmid enrichment

Yu et al. showed that a plasmid enriched with 24 CpG motifs (pBISIA24-NS3) tends to induce a strongest and consistent Th1-biased immune response. Subsequently, it was shown that NS3 formulated with CpG oligodeoxynucleotide and Quil A (rNS3 + CpG + Quil A) adjuvants induces a balanced immune response in mice, compared to recombinant NS3 combined with either CpG or QuilA which elicit a Th2-biased response. To further enhance NS3-specific cell-mediated immune responses, a vaccination regime consisting of priming with pBISIA24-NS3, followed by boosting with rNS3 + CpG + Quil A, was explored in mice and pigs. In contrast to immunization with rNS3 + CpG + Quil A, this regimen shifted the immune response to a Th1-type response and, accordingly, enhanced MHC I-restricted killing by CTL in mice. Although immunization with pBISIA24-NS3 also induced a Th1-biased response, including cytotoxicity in the mice, the humoral response was significantly lower than that induced by the DNA prime–protein boost regime [59].

### Utilization of co/post translational modification

In a recent study by Masavuli et al. incorporation of secreted E1 and E2 (sE1 and sE2) into oligomers by fusion with the oligomerization domain of the C4b-binding protein, IMX313P resulted in increased effectiveness in eliciting humoral and cell-mediated immunity against the envelope proteins. Further boosting with recombinant E1E2 proteins but not DNA nor virus-like particles expressing E1E2 increased the immunogenicity of the DNA prime-boost regimen. However, antibodies generated by the homologous DNA prime-boost vaccinations more effectively inhibited the binding of virus-like particles to target cells and neutralized transduction with HCV pseudoparticles derived from different genotypes including genotypes 1, 2, 3, 4, 5, and 6 [60].

Analysis of immunogenicity of wild type E1E2, five N-glycosylation sites-mutated E1E2 glycoproteins, and five CpG-coupled E1E2 N-glycosylation-mutated glycoproteins in BALB/c mice showed that deletion of N-glycans can enhance viral immunogenicity and the CpG-coupled DNA vaccine mutant elicited increased CD4+ Th1 and CD8+ T cell responses and neutralizing antibody production against HCV infection [61].

Modification of N-linked glycosylation of HCV E1 protein, a naturally poor immunogen, through site-directed mutagenesis of N-linked glycosylation sites in plasmids containing the genes for both wild type and mutant E1 in BALB/C mice resulted in enhanced E1-specific CTL activities, expression of IFN-γ producing T cells, and suppression of tumor growth E1-M2 mutant (at site of N209SS). While E1-M4 mutant (at site of N305CS) induced the highest specific antibody response among all groups. Moreover, E1 wild-type vaccinated mice developed a mixture of IgG1 and Ig2a, but E1-M2 mutant induced only IgG2a isotype, and E1-M4 mutant dominantly developed IgG1 isotype. According to the study, N-linked glycosylation can limit both cellular and antibody response to the HCV E1 protein and deletion of the glycosylation sites at N209SS and N305CS in the envelope protein E1 results in higher immunogenicity [62].

In addition, immunization with plasmids containing genes encoding either wild type or mutant E2 proteins with mutated N-glycosylation sites (N560NT and N576ST) close to these regions were mutated separately or in combination led to significantly higher E2-specific CTL response, IgG2a/IgG1 ratios, expression of IFN-γ, and suppression of tumor growth (P < 0.05) with the E2-M2 mutant (at N576ST) compared to control. IgG2a/IgG1 ratios were elicited in a Th1-type response. Therefore, modulation of the N-glycosylation site N576ST of HCV E2 protein may enhance specific cellular immune response and could be utilized in the development of E2-based DNA vaccines with enhanced immunogenicity [63].

## Discussion

Successful induction of immune response against transduced antigen proteins in the early 1990s introduced the concept of DNA immunization into the scientific spotlight [64]. Nucleic acid vaccines have been proven since then to be a safe platform to elicit protective humoral and cellular immune response to a variety of infectious agents and diseases as well as therapeutic modalities to treat malignancies and autoimmune disorders [64–66].

Compared to classic non-live vaccines, nucleic acid-based vaccines could be easily mass produced and can readily stimulate humoral and, particularly cellular immune responses in preclinical studies, which is favorable for immunization against viral infections such as HCV. Furthermore, they do not carry the risk of transmitting active infection in immunocompromised individuals as seen with live attenuated vaccines while similarly mimicking live infection [64, 67]. As such, DNA vaccines would be optimal candidates to induce strong and protective CD4 + T-cell and CTL response early on in HCV infections, where viral clearance is closely associated with virus-specific T cell immunity [68, 69]. Nevertheless, the suboptimal immunogenicity and anti-vector immunity observed in first generation DNA vaccines in primates has highlighted the need for strategies to enhance the immune response as described in this review [51].

While this systematic review attempted to cover studies on RNA vaccines in HCV, the initial database search revealed no studies fitting the inclusion and exclusion criteria, reflecting the lack of adoption of widespread RNA platform in vaccine HCV design. Although the initial concerns regarding instability and large-scale manufacturing of RNA halted the widespread adoption of RNA vaccines, the promising aspects of this type of vaccines such as the ability to express a variety of antigens with high efficiency while having no risk of integrating exogenous DNA into host genome, has made them an attractive alternative to DNA vaccines [67, 70]. The preliminary reports on mass vaccination with SARS-CoV-2 mRNA vaccines have demonstrated their general safety and high efficacy in disease prevention [71]. Therefore, with proper backbone, mRNAs encoding HCV antigens [72] could obviate the need for electroporation to generate sufficient immune response and demonstrate higher efficacy and immunogenicity compared to DNA vaccines.

This review summarized recent original researches on the topic of HCV DNA vaccines and discussed the strategies on optimization of HCV DNA vaccines and their efficiency. Based on the reviewed literature, the authors suggest that incorporation of multiple viral proteins or their epitopes in a homologous or heterologous prime-boost regimen or using CpG-enriched DNA and recombinant virus vaccines with coexpression of potent adjuvants with high immunogenicity are valid strategies to increase the potency of potential vaccines. Considering the limited efficacy of the results of the registered clinical trials with candidate vaccines [73, 74], future studies show focus on maximizing the immunogenicity of their vaccines by incorporating the strategies mentioned throughout this text.

## Data Availability

The datasets supporting the conclusions of this article are included within the article.

## Abbreviations

HCV: Hepatitis C virus
CTL: Cytotoxic T lymphocyte
HBV: Hepatitis B virus
IL: Interleukin
PRF: Perforin
CCL20: CC-chemokine ligand 20
GM-CSF: Granulocyte-monocyte colony stimulating factor
NS: Hepatitis C non-structural protein
IFN-γ: Interferon-γ
ELISpot: Interferon-γ enzyme-linked immunosorbent spot
Th: T helper
